# CURA: A clinical ethics support instrument for caregivers in palliative care

**DOI:** 10.1177/09697330221074014

**Published:** 2022-05-27

**Authors:** Suzanne Metselaar, Malene van Schaik, Guy Widdershoven, H. Roeline Pasman

**Affiliations:** Department of Ethics, Law & Humanities, 1209 Amsterdam University Medical Centers, Amsterdam, The Netherlands; Department of Public and Occupational Health, 1209 Amsterdam University Medical Centers, Amsterdam, The Netherlands

**Keywords:** clinical ethics support, palliative care, new interventions, moral resilience, moral competences

## Abstract

This article presents an ethics support instrument for healthcare professionals called CURA. It is designed with a focus on and together with nurses and nurse assistants in palliative care. First, we shortly go into the background and the development study of the instrument. Next, we describe the four steps CURA prescribes for ethical reflection: (1) Concentrate, (2) Unrush, (3) Reflect, and (4) Act. In order to demonstrate how CURA can structure a moral reflection among caregivers, we discuss how a case was discussed with CURA at a psychogeriatric ward of an elderly care home. Furthermore, we go into some considerations regarding the use of the instrument in clinical practice. Finally, we focus on the need for further research on the effectiveness and implementation of CURA.

## Introduction

Although giving palliative care to patients is generally experienced as very rewarding by many professionals,^1,2^ some palliative care situations may be experienced as morally troublesome.^3–7^ In these situations, moral conflicts may arise. In the case of a moral conflict, values or obligations that matter in the situation are at odds with each other. Moral conflicts arise either when others involved in the situation (such as the patient, a family member, or a colleague) think differently about what constitutes “good care” in that situation, or when someone experiences an internal conflict between the norms and values that (implicitly) guide them in their daily work.^8,9^ In palliative care, moral conflicts may, for instance, arise from different perspectives on whether to continue life-prolonging interventions or not, or from having to choose between adhering to protocols and guidelines or diverging from them in order to meet a patient’s wishes at the end of life.^10^

These moral conflicts may deeply impact health care professionals and may cause *moral distress*, that is, psychological distress in relation to a moral event.^11,12^ When moral distress remains unresolved, it may lead to exhaustion, feelings of disengagement, depersonalization, and numbness.^13–15^ Furthermore, moral distress is associated with decreased job satisfaction,^16^ lower quality of care,^17,18^ and burn-out.^19^

Levels of moral distress are found to be high in palliative care, in particular in the context of generalistic health care settings.^10^ Nurses are found to experience higher levels of moral distress than physicians.^10,20,21^

Considering the above, it is important that professional caregivers, especially nurses, are able to deal well with the moral conflicts they experience in palliative care practice. Their ability to do so can be strengthened by building *moral competences*: skills and qualities such as a reflective attitude, being able to identify and articulate ethical challenges, exploring and understanding the moral perspectives of other stakeholders in the situation, and coming to principled actions based on careful deliberation.^8,22,23^ These moral competences are argued to be important for the quality of (palliative) care *itself*.^6,15,17,18^ Furthermore, developing moral competences is argued to foster “moral resilience,” that is, being able to deal with and overcome moral distress, which is essential to the well-being of care professionals.^14^

To support professionals in dealing with moral conflicts and to train their moral competences, clinical ethics support services (CESS) are offered. CESS address moral conflicts in clinical settings, often by fostering reflection among professionals on their own moral dilemmas in health care practice.^8,24–26^ For this purpose, clinical ethics support instruments (CES instruments) have been developed, that is, tools and methods that provide guidance in dealing with moral issues in clinical practice, for instance by methodically structuring a joint reflection process.^27^

Different ethics support instruments already exist, such as Moral Case Deliberation,^28^ the Nijmegen Method of ethical case deliberation^29^ or METAP.^30^ The feasibility and outcomes of (some of) these instruments have been studied,^31–34^ and they are found to improve caregivers’ moral competences and to reduce moral distress.^23,31,35^

However, existing CES instruments are not always optimally tailored to specific health care settings, and are found to have limitations. For instance, with regard to Moral Case Deliberation, caregivers report that clinical ethics support sessions are often time-consuming, as they may take between one and 2 hours, making it difficult to implement them in daily routines. Furthermore, many existing instruments require the guidance of a trained facilitator or ethicist due to their complexity. This guidance is not always available when an urgent situation occurs.^36,37^

Therefore, in the context of a large national programme to improve the quality of palliative care in The Netherlands, we developed CURA, a four-step CES instrument to support health care professionals in dealing with their moral conflicts in palliative care practice. By tailoring it to the needs and wishes of professionals working in various palliative care settings in the Netherlands, we sought to overcome the aforementioned limitations. In this article, we present this instrument. First, we will shortly go into the way in which CURA was developed in close collaboration with stakeholders from practice. Subsequently, we will describe the four steps of CURA, and present a case discussion in order to illustrate the way in which CURA is used (Box 1). Subsequently, we provide some considerations on the use of the instrument. Finally, we provide an outlook on further research. The instrument itself is added as an Appendix to this article.

### Development of the instrument

CURA is the result of a 2-year study on the basis of a participatory development design: a participatory development study involves close collaboration between researchers and participants, departing from the idea that, if you want to create useable services or instruments, you should involve the people who are going to have to use them, as well as other important stakeholders.

Reflect

First, the participants ventured into the perspectives of those involved and thought about what might have been important to them. For Mrs. A., they argued, it might be important that her boundaries and bodily integrity are respected, that her wishes are acknowledged, and that she does not have to undergo activities against her will. It might also be important for her to be comfortable, and not anxious. For the *family members*, it might be important that their mother is both calm and clean. Furthermore, they indicated that they do not want their mother to be a burden to the health care providers. The *physician* is responsible for a safe environment, for both the client and the staff, the participants argued. Hence, (taking responsibility for) safety could be a concern for her. Furthermore, deciding on a treatment plan that all parties can agree with might be important for the physician as well. For the *colleagues* of the nurse who were involved in showering Mrs. A., “good care” is important, meaning that clients are washed. The nurse explains that, in this care home, caregivers take great pride in their clients being fresh and well-dressed. Furthermore, safety is be important, and that the client is at ease and not in any distress.

The nurse who presented the case indicated it is important to her that she is able to constantly attune to the needs of the client while providing care. In this case, this means being able to connect and communicate with Mrs. A. while washing her. Sedating Mrs. A. makes her loose this connection. Subsequently, a brief inventory of relevant laws, protocols, and guidelines is made. The Dutch Care and Coercion Act for psychogeriatric and mentally disabled clients (2019) is mentioned. This law aims to limit coercion and involuntary care as much as possible, meaning that it is only allowed when no other alternatives are feasible and when there is a risk of “serious harm” for either the client and/or caregivers. The participants agreed to look into the details of this law afterward. The participants also wondered whether there would be alternatives that could be tried, such as washing her with washcloths rather than in the shower, and how the client’s family would feel about this.

Act

After carefully considering what had been discussed thus far, the participants considered for themselves what should be prioritized in taking action. After having a dialogue on their considerations, the nurse came to conclude that for her, “good care” first and foremost implies being able to connect to the client, and being able to attend to her caring needs. In this case, she argued, these needs are primarily not being forced into a situation that causes great anxiety and being as comfortable as possible. Also, she cannot properly connect to Mrs. A. when she is sedated. Therefore, she decided that she will talk to the physician, her colleagues and, if possible, to the family. She intended to express the reasons for her hesitation to use sedatives, and to discuss whether there are other ways to wash Mrs. A., for instance at the basin with washcloths. This action is in accordance with what she stands for as a professional, she argued, because she strongly believes that attentiveness to the client’s needs is essential for good care.

The participants ended with evaluating the process. The nurse mentioned she feels supported and relieved. She no longer felt “unfit” for her profession because of her doubts. Furthermore, she realized that her colleagues also wanted to provide “good care,” but have a different understanding of what this entails. This helps her to be more engaged with the team, she concluded.

The following week, the nurse told the researcher that, after a conversation with the physician and her colleagues, her colleagues also became less comfortable with giving Mrs. A. sedatives and showering her. The team agreed not to do this any longer, and to look for alternative ways to wash Mrs. A., and to discuss this with the family.

The benefits of a participatory approach to the development of a health care intervention—in this case a CES instrument—is that stakeholders’ wishes, needs, and demands can be taken into account from the very beginning of the development process, which promotes the feasibility and acceptability of the intervention.^38^ It also promotes co-ownership and responsibility among the intended users, and thus it supports implementation of the intervention.^39^ Furthermore, the development process optimally benefits from the (experiential) knowledge of stakeholders. For instance, with regard to what would best fit the care setting(s) in which the intervention is intended to be used.^38^

We developed CURA together with a so-called *community of practice* (CoP), that is, a group of people sharing a common interest or goal that can develop new forms of practice.^40^ The members of the CoP consisted of nurses and other caregivers (the envisioned primary end users of CURA), educators and trainers, implementation experts, managers, palliative care consultants, patient representatives, and representatives of volunteer organizations.

The development process consisted of four phases. In phase one, user needs and preferences were identified: the members of the CoP indicated which characteristics the instrument should and should not have, on the basis of which we established a very first concept. Phase two focused on further developing and redesigning the instrument on the basis of an iterative process of co-creation. We did four rounds of testing in practical and educational settings. After each round, we evaluated and adapted the instrument. Phase three focused on disseminating the finalized instrument, now called “CURA.” Among other things, we organized a national conference in which CURA was introduced to a wider audience, and in which frontrunners shared their experiences. The fourth phase focused on joint evaluation of the process and making plans for implementation in both in health care and educational settings.

Both qualitative and quantitative methods for data collection were used. Participative observation was used during try-out sessions and the CoP work sessions were carefully documented. Two focus groups with caregivers were organized to discuss the feasibility and implementation of CURA in practice. Furthermore, a questionnaire on CURA’s feasibility and perceived effects was distributed (*n*=221). Respondents found CURA accessible and easy to use, its steps being clear, and found that CURA was helpful in dealing with the moral challenges they encountered in their work. However, respondents indicated that they would need (more) organizational support (especially, allocated time) for optimal use of CURA in daily practice.^41^

## The instrument

The name CURA was chosen for several reasons. First of all, the Latin word *cura* refers to both the practice of *caregiving* as well as to *concern* whether the care that is given is indeed the right kind of care in the situation at hand. This points towards the fact that caregiving and ethical considerations—is this indeed *good* care?—are intrinsically connected. Second, the name “CURA” refers to the goddess of care, “Cura,” as described in an allegorical story of Hyginus (ca. 7 AD). Third, CURA is an acronym, referring to the four steps of the moral reasoning process it seeks to foster: 1) Concentrate, (2) Unrush, (3) Reflect, and (4) Act.

In the first step, Concentrate, users focus on elucidating the details of the situation that is experienced as morally troublesome and carefully articulating their moral doubts regarding “good care” in that specific situation. In the second step, Unrush, users explore their emotions and initial judgments of the situation. The third step, Reflect, focuses on examining the moral perspectives of those involved in the situation, and on relating these perspectives to what relevant guidelines, protocols, or legislation prescribe. In the fourth step, Act, users reflect on what they consider to be *most* important, and decide what should be leading in taking principled actions. In the following, we will elaborate on these four steps. Complementary to this hand-out, we developed a manual in which we provide more elaborate instructions and guidelines for users.

Step 1: Concentrate

The aim of this first step is to *concentrate* on the situation and their experience of it as morally difficult or questionable. The first question is:

• *Take a moment to reflect on the situation. Describe this situation briefly.*

One participant, the “case presenter,” describes the actual situation based on his or her experience (who, what, when, where, and how), refraining from moral judgments and hypothetical considerations, and withholding conclusions on how to deal with the situation. Significant details should not be omitted in elucidating the situation, however, being too broad should be avoided. Other participants may ask questions in order to further clarify the case, but should not address normative aspects, or bring in their own experiences, judgments or solutions yet. Subsequently, the case presenter is asked to articulate his or her moral doubts with regard to the situation:

• *What are your doubts concerning good (palliative) care?*

Importantly, this doubt should be a *moral* doubt, that is, a doubt concerning the right thing to do in order to promote “good care” in the situation at hand (rather than a doubt that is primarily of a medical, legal, or technical nature). This doubt may be articulated in different ways. For instance, it may be phrased as a *moral dilemma*, that is, an actual choice between two courses of action that could be described as a choice between “two evils.” It may also be formulated in a more general way, such as “What should we do in order to provide good (palliative) care to this patient in this situation?.” In any case, it is conducive for the quality of the deliberation when the case presenter describes his or her doubt as precisely as possible.

Step 2: Unrush

The central aim of this step is to become aware of one’s *initial* cognitive, emotional, and physical responses to the situation. Situations that are experienced as morally difficult often evoke strong first judgments and emotions, sometimes accompanied by bodily reactions (for instance, nausea, numbness, and a headache). This is understandable, as health care is a practice in which caregivers are bodily, socially, and emotionally immersed. Although the value and role of emotions are recognized in some approaches to clinical ethics support,^42^ most CES instruments do not explicitly and/or methodically pay attention to emotions. Nonetheless, addressing them is important in dealing well with moral issues. First of all, becoming attentively aware of these negative emotions is conducive to dealing well with moral distress and to building moral resilience.^14^ Second, paying attention to emotions is important because of their moral value; we can learn from our emotions about what is truly important or worthy of protection to us in a given situation.^42,43^ Finally, becoming aware of your initial response to a moral conflict may help you to *postpone* or critically explore a prejudice, allowing for a more open mind when venturing into the moral positions of other parties involved.^44^

Therefore, participants are asked to perform the following actions:

• *Identify your initial reaction to the situation and to those involved (first judgments and emotions).*
• *“Park” your initial reaction for a while so that you can explore the situation with an open mind.*

In a joint deliberation, it is important that participants have a dialogue on the above, and take some time to listen to each other without judgment.

Step 3: Reflect

The aim of this step is to broaden one’s moral perspective on the situation and gaining new insights by means of exploring what is important in the situation according to *whom* (the patient, relatives, colleagues, and yourself) and *what* (protocols, laws, and guidelines). The former pertains to the unique moral perspectives of individuals immersed in the situation, whereas the latter pertains to a more general ‘impersonal’ moral perspective related to the issue at hand.

First, this step is about examining moral perspectives of stakeholders, starting with the patient. Participants are also invited to consider what is of value for themselves:

• *What is important in this situation?*

For the patient

For others involved ( i.e. relatives, colleagues, etc.)

For you

Essentially, this question inquires after people’s values, norms and principles, that is, what people deem important, what they strive for and live by, although participants do not need to distinguish between these categories. Participants should focus on what is relevant to the situation at hand. This exploration can provide new perspectives on the case that lead to a deepened an enriched understanding of what is valuable in the situation. If moral perspectives are explored of people who are not present during the reflection, the group should focus on reconstructing their perspective as good as possible. They can do so by putting themselves in their shoes, and by departing from what they know about them.

The next question inquires after general moral perspectives on the situation as embodied in laws, protocols, and guidelines:

• *What do laws, protocols or guidelines say?*

Discussing what is known to the participants at that particular moment is sufficient, as searching for relevant legislation, protocols and guidelines might be too time-consuming, and distract from the reflection. This can be done either beforehand or afterward.

Finally, there might be things still unknown or uncertain to the participants that might be relevant to the reflection. Becoming aware of these lacunas may prevent making incorrect or incomplete assumptions, for instance about what is important to someone who is not present. Hence, the final question of this step is focused on the identification of uncertainties or lacunas in understanding the situation, which might be further explored afterward:

• *What is still unknown or uncertain to you?*

Step 4: Act

The fourth and final step of CURA focuses on what to do in the difficult situation at hand, whereas the former step focused on exploring what is important from various perspectives, this step aims at weighing and balancing what has emerged, and establishing what is *most* important. It is about *moral judgment in relation to taking action*, that is, deciding what is the right thing to do based on a careful consideration and prioritization of all that morally matters in the situation. This requires a “balancing act” not far removed from the way in which Beauchamp and Childress describe making balanced judgments, which always involves “some intuitive and subjective weightings (…) just as they are everywhere in life when we must balance competing goods.” At the same time, they insist that “balancing (…) is a process of justification only if adequate reasons are presented.” (Beauchamp and Childress, 34–36). In this step, participants are therefore asked:

• *What do you find* most *important in this situation?*

Ideally, participants can also explain why they prioritize certain values or considerations over others. For this process, participants need to take some time. Intrinsically connected to the prioritization of what is most important is the question *how to act upon this*. Hence the question:

• *On this basis, what are you going to do?*

It needs to be noted that “doing” is not always a question of rolling up your sleeves: sometimes “taking action” just means speaking up, initiating a conversation, or even accepting the situation as it is. Whatever decision is made, it is wise here to consider what possible negative consequences your chosen line of action may have, and how these can be prevented or limited.

This step does not explicitly inquire after how to reconcile differences in perspectives among the participants of the reflection process. However, sharing and comparing moral judgments and engaging in a dialogue is recommended. In some cases, consensus might be necessary in order to deal with the situation in a good way, for instance if the participants are all part of the same treatment team, whereas in other cases, exploring differences in judgments on what should be action-guiding in the situation may encourage moral learning—even when differences remain.

The third question of this step is:

• *How does this relate to what you stand for as a professional?*

In order to answer this question, participants can consider their motives for working in palliative care, and establish whether and how their choice for a course of action in this particular situation relates to these motives. Dealing with this question is not strictly necessary in dealing with the moral conflict at hand. However, research shows that consciously acting according to what you stand for may help you in dealing with moral distress in your work.^45^ Cynda Rushton describes this as “moral integrity,” that is, consistently aligning one’s actions with one’s ethical values, especially in times of adversity.^46^ This allows you to act in line with what you deem important even if the situation does not evolve the way you would have wanted it to.

The final questions of this last step of CURA focus on the evaluation of both process and outcomes. Which insights were gained? Has your moral understanding of the situation changed? Have your feelings towards the situation changed? Has your moral doubt diminished?

• *Have you gained new insights?*
• *Have your feelings about the situation changed?*

If your doubts and/or negative emotions have decreased, this may be an indication that you have found a good way to deal with the situation, and/or with the emotional burden or moral distress that the situation brings about. However, it may be the case that you come to conclude here that you have not yet sufficiently dealt with the moral issue at hand, and that you want to involve another resource or party, such as an ethicist, ethics committee, and/or organize a different kind of ethical reflection. In the box below, we give an example of the way in which CURA is used in practice.

**Box 1: A case example**

The following reflection with CURA took place among two nurse assistants and one nurse, all working in a nursing home. They all had some previous experience with using CURA. The researcher (MvS) observed and made field notes—but did not take part in the reflection herself. The participants gave both written and verbal consent for the use of this case for academic purposes. We adjusted some details of the case in order to warrant the privacy of those involved.

**Concentrate**

The nurse presented the following case:

Mrs. A. is an 80-year-old woman who has been living in the nursing home for years. Some time ago, she moved to the psychogeriatric ward because her Alzheimer’s disease had progressed. She now needs 24/7 specialized care. On the ward, clients are showered two times a week. However, due to a traumatic experience as a child, Mrs. A. is afraid of water. Because of this trauma, she always used to wash herself at the sink. But now, the nurse explains, “we need to wash her, as she no longer can do this herself. My colleagues want to shower her, just like we do with the other clients. But every time, this is horrible. She strongly resists, panics, and strikes, scratches and kicks us. Her daughter and son), her physician and my colleagues discussed the situation and came to a shared decision: we will shower her once a week and give her sedatives beforehand. Consequently, she will not be anxious and we can do our work safely and calmly. However, I am in doubt: is this really good care for Mrs. A?”

Subsequently, the two other participants asked questions to gain a better understanding of the situation. For instance, they asked what the relationship between Mrs. A and her family members were like. According to the nurse presenting the case, they have a good relationship and the family is very involved.

**Unrush**

In this step, the participants shared their initial response to the situation. Some of the reactions they expressed were: “I feel sad for the client” (nurse assistant 1). “It gives me a feeling of disengagement and cynicism. We might as well sedate all our clients” (nurse). “On the other hand, I wouldn’t want to get a black eye. Our safety is important.” (nurse assistant 2). “I feel physical unease, I want to walk away from the situation.” (nurse). Furthermore, the nurse maintained: “I guess I am probably not fit to work in healthcare. My colleagues, the physician and the family all think this is a good idea. Am I the one who’s crazy?.” Sharing these first responses created a space for the participants, especially for the nurse who presented the case, to acknowledge and share their emotions and doubts concerning the situation. Also, they found that their initial reactions gave them a first indication of what is important to them, such as the staff’s safety, and the client’s well-being. Finally, the participants made a conscious effort to postpone these initial responses in order enter the next step of the reflection with an open mind.

## Using CURA: Some considerations

We would like to address some considerations with regard to the use of CURA. These considerations concern preconditions for proper use of CURA in palliative care practice as a CES instrument as well as some instructions as they emerged from our development study.

First of all, CURA may be used either prospectively, that is, in anticipation of making a decision in a morally difficult situation, or in retrospect, that is, looking back at that situation. In the former case, CURA may contribute to making a well-considered choice for a course of action. In the latter case, CURA may be used to reflect on a situation in the (recent) past, especially when doubts are still lingering as to whether the moral conflict experienced in that situation was dealt with in a good way. This may offer valuable lessons for future situations,^47,48^ and help caregivers in dealing with the aftermath of the situation.^49^

Second, CURA is developed to be used either individually or jointly. In the first case, CURA might help to structure your thoughts, articulate your moral doubts, to become aware of the way in which a situation affected you, of what is important to you and to others involved, and to make up your mind about how to deal with the situation. It is, however, preferable to go through the reflection process together with others, because engaging in a reflective dialogue on a moral problem fosters moral learning. For one thing, it might broaden one’s perspective on the situation as other participants might add valuable knowledge about the situation or points of view you did not yet take into consideration.^47,48^

Third, CURA is developed as a low-threshold CES instrument; no formal training is necessary to initiate, lead or participate in a reflection with CURA. To provide support in properly using CURA, either as moderator or as participants, we developed materials such as a manual and e-book that offer instructions and context.

Fourth, when using CURA jointly, we do advise appointing to one of the participants the role of moderator or “facilitator” of the reflection with CURA. The moderator may (1) ensure that the group goes through all steps of the method in the right way and order, (2) manage the time available, (3) encourage a dialogical attitude of the participants, and (4) help to focus on an in-depth reflection on the case at hand, and not diverge into other situations or subjects.

Fifth, we sought to develop an instrument that could be used—after users have gained some experience with it—in approximately 30–45 min, as stakeholders participating in our CoP told us that this time frame makes it easier to embed CURA in care settings in which time for reflection is often scarce, and to promote the integration of ethical reflection in existing meetings. Yet, studying the use of CURA in palliative care practice, we found that people sometimes need more time, for instance in complex cases, when a larger group of caregivers took part in the reflection, and/or when participants are new to CURA. Hence, this time frame is merely an indication, and should not dictate the length of the reflection.^50^

As we designed CURA as a relatively simple method for ethical reflection among health care professionals that can be easily embedded in daily work routines, we recommend to use it in a relatively small group (2–6 participants) in an informal setting. CURA can be used with larger groups in a more formal setting. However, this asks for additional skills, most of all from the facilitator of the reflection, as well as more time.

Sixth, we would like to stress that merely following a series of steps in order to structure an ethical reflection is not a panacea for a *good* ethical reflection. We therefore recommend CURA users to take on a dialogical and reflective attitude, such as postponing your judgments and ideas for actions, critically exploring your own thoughts, actively putting yourself in the position of others, asking questions and listening rather than trying to convince others of your own point of view.^8,47^

Finally, we would like to stress that merely following a series of steps in order to structure an ethical reflection is not a panacea for a *good* ethical reflection. We therefore recommend CURA users to take on a dialogical and reflective attitude (Table 1). In addition, we recommend users to take recourse to other forms of ethics support (consulting an ethicist, a clinical ethics committee, or using another CES instrument) if the reflection with CURA is experienced as insufficient to deal with the moral issue at hand.

## Discussion and conclusion

In this article, we presented CURA, a CES instrument developed with and for health care professionals in palliative care. With CURA, we sought to develop an instrument that is feasible in every day practice, taking into account the preferences and needs of end users and other stakeholders. CURA presents a relatively simple method for ethical reflection, and can be used without elaborate training within a relatively short time-frame (ca. 30–45 min). Materials with instructions, examples, and best practices are available online.

CURA differs from other CES instruments (as mentioned in the Introduction) because (a) of its simple structure (four steps), (b) it can be used within a short time frame (30–45 min Is indicated), (c) it pays explicit attention to embodied experience (emotions, physical response, in the step “Unrush”). From our research up until now, we observe that these specific characteristics of CURA have the following benefits: (1) the simple structure makes CURA easily accessible and useable for a variety of caregivers, also because they do not need to engage an extensively trained facilitator or ethicist, (2) it is easier to integrate CURA in daily work routines—in which time is often scarce, (3) paying attention to emotional responses to a situation that is experienced as morally troublesome is considered to be important by caregivers in dealing well with the burden (moral distress) that may result from moral conflict.^13^

These observations are reflected in the results of our feasibility study. This study has shown that nurses perceive CURA to be easy to use and helpful in building moral competences such as perspective taking and articulating their moral challenges. It is also experienced to reduce moral distress.^41^ However, more research is needed to assess the effectiveness of CURA and its feasibility in different health care and educational contexts. For instance, does CURA result in moral resilience and/or reduce moral distress in the long term? Does it foster the moral competences of health care professionals who use it on a structural basis? A 3-year mixed-method study in 10 Dutch health care organizations, which we started in 2020, aims to provide us with more insight on CURA’s effects. In order to measure the effect on professionals’ moral competences, we use the European Moral Case Deliberation (Euro-MCD) Scale.^35^ In order to measure CURA’s effect on moral resilience, we use the Rushton Moral Resilience Scale (RMRS), of which we made a translation into Dutch as well as a context validation for the Dutch health care setting.^51,52^ In order to gain a deeper understanding of the effects of CURA, we combine these scales with qualitative research (interviews and focus groups).

Furthermore, research is needed to gain insight in how CURA can best be implemented in various health care settings. What are organizational preconditions? What are strategies to overcome obstacles in using CURA on a structural basis? Parallel to our effectivity study, we therefore started an implementation study in the same 10 organizations. In the context of this implementation study, we therefore developed a blended learning training program in order to train so-called “CURA-ambassadors.” These ambassadors are to introduce, initiate, and facilitate ethics support sessions with CURA, and thus contribute to its implementation within their organization.

As CURA was developed in the context of a national programme that seeks to improve the quality of palliative care, our focus was on developing ethics support specifically for palliative care professionals, especially nurses. During our study, we observed that CURA is used for a wide variety of palliative care cases, ranging from moral dilemmas in dealing with wishes of the patient or family, treatment decisions, collaboration with other professionals, to dilemmas related to physician-assisted dying. In addition, CURA was frequently used to reflect on COVID-19 cases, especially with regard to end-of-life situations and difficult treatment decisions. We notice that users tend to focus on individual patient cases when using CURA, rather than, for instance, on more encompassing (policy) issues.

However, CURA was also positively received by other healthcare professionals, such physicians and volunteers, and is now used in other domains of health care as well. Recently, the Dutch syndicate for nurses and nurse assistants has adopted CURA as a method for ethical reflection they recommend to their members in *all* care settings, and we have been collaborating with them in adapting our materials for this purpose. However, more research is needed to assess the feasibility and effectivity of CURA for purposes beyond providing ethics support to professionals in palliative care. In addition, it is key to explore how CURA can be best implemented in education: as many participants in our research indicated, it is important that health care professionals are trained in ethical reflection during their training in order to prepare them for dealing with moral conflicts in their work.^53^

Finally, an issue that is both fundamental and challenging pertains to the quality of ethical reflection obtained with CURA. CURA fosters *ethical* reflection on a case rather than technical or medical reflection. It asks to articulate one’s doubts about “good care,” and to reflect on what is *important* to those involved. The latter request is a relatively simple and concrete way of asking after the values, norms, and principles that underlie perceptions of “good care” in the situation at hand. The instructions on the CURA handout (Appendix to this article) and a manual with more elaborate instructions (not included here) further steer the reflection towards considering specifically the moral dimensions of the case.

However, does CURA warrant that an ethical issue is explored in sufficient depth? Does it provide sufficient support for professionals to deal with their moral doubts in a good way? Does it lead to better (palliative) care? Answering these questions is dependent on normative assumptions about what constitutes “good ethical reflection,” “good care,” etc. These assumptions would have to be made explicit first, before this is empirically studied.^26^ Another issue is how CURA relates to other CES instruments: can or should CURA be used complementary to other approaches to clinical ethics support, or to consulting an ethics committee?

To conclude, CURA is a promising four-step CES instrument, which we developed together with and for healthcare professionals working in palliative care. The instrument is relatively simple and can be used in a relatively short time frame. It is developed to foster ethical reflection on everyday care by health professionals themselves, preferably in informal, smaller groups. Further research is needed, for instance to assess the effectiveness of CURA in fostering moral competence and moral resilience.
